# Incongruent gestures slow the processing of facial expressions in university students with social anxiety

**DOI:** 10.3389/fpsyg.2023.1199537

**Published:** 2023-08-22

**Authors:** Xinyi Zhu, Yan Gong, Tingting Xu, Wen Lian, Shuhui Xu, Lu Fan

**Affiliations:** ^1^Department of Psychology, School of Education, Wenzhou University, Wenzhou, China; ^2^Department of Psychology, Jing Hengyi School of Education, Hangzhou Normal University, Hangzhou, China

**Keywords:** social anxiety, facial expression, gesture, attentional bias, context effect

## Abstract

In recent years, an increasing number of studies have examined the mechanisms underlying nonverbal emotional information processing in people with high social anxiety (HSA). However, most of these studies have focused on the processing of facial expressions, and there has been scarce research on gesture or even face-gesture combined processing in HSA individuals. The present study explored the processing characteristics and mechanism of the interaction between gestures and facial expressions in people with HSA and low social anxiety (LSA). The present study recruited university students as participants and used the Liebowitz Social Anxiety Scale scores to distinguish the HSA and LSA groups. We used a 2 (group: HSA and LSA) × 2 (emotion valence: positive, negative) × 2 (task: face, gesture) multifactor mixed design, and videos of a single face or gesture and combined face-gesture cues were used as stimuli. We found that (1) there is a distinction in the processing of faces and gestures, with individuals recognizing gestures faster than faces; (2) there is an attentional enhancement in the processing of gestures, particularly for negative gestures; and (3) when the emotional valence of faces and gestures align, it facilitates the recognition of both. However, incongruent gestures have a stronger impact on the processing of facial expressions compared to facial expressions themselves, suggesting that the processing of facial emotions is more influenced by environmental cues provided by gestures. These findings indicated that gestures played an important role in emotional processing, and facial emotional processing was more dependent on the environmental cues derived from gestures, which helps to clarify the reasons for biases in the interpretation of emotional information in people with HSA.

## 1. Introduction

Social anxiety (SA) refers to anxiety in response to interpersonal communication with others (Pierce, 2009; Ran et al., 2018). People with high SA (HSA) have persistent fear and avoidance of social settings and cannot easily communicate with their peers (Stein and Stein, 2008). In recent years, studies have shown that social anxiety disorder (SAD) is one of the most frequently diagnosed anxiety disorders (Crome and Baillie, 2015; McBride et al., 2022) after major depressive disorder and alcohol dependence, with a greater risk of prevalence in adolescents (Jefferies and Ungar, 2020). Individuals with HSA are prone to bias in some stages of information processing. For example, in the early stage of information processing, they are more likely to shift their attention to threatening stimuli (Schmidt et al., 2009) and find it difficult to disengage from threatening stimuli (Moriya and Tanno, 2011). This feature of attention fixation and attention enhancement is called attentional bias (Atkinson et al., 2004). The cognitive model theory of SA holds that this attentional bias toward negative or threatening stimuli is not only a key process that leads to anxiety (Mogg and Bradley, 2018) but also underlies the persistence of symptoms in people with HSA (Heimberg et al., 2010; Jiang et al., 2019).

In our daily life, recognition, understanding and expression emotions are key to human social communication. Emotion recognition refers to the ability of humans to identify emotional states, which is an inherent multimodal phenomenon that is based on different cues (e.g., verbal, facial, body posture or vocal cues) that are arranged in various patterns (e.g., visual, auditory or multichannel processing) (Atkinson et al., 2004; Matsumoto and Hwang, 2014). In addition to facial expressions, body expressions are a major source of information for identifying an individual’s emotional state (De Gelder, 2006; de Gelder et al., 2014; Borhani et al., 2015; Bachmann et al., 2020). Research has found that there may be similar mechanisms between individual processing of the body and facial expression processing (Ding et al., 2017, 2018). In real life, facial expressions do not exist alone but are often accompanied by corresponding body movements, scenes, voice and intonation. These cues can also help us to identify emotions effectively. Compared to other emotional cues, body expressions can provide us with more realistic emotional information and help us identify the emotional state of others. In real life, people receive more combined physical and facial stimulation. Studies on the interaction between face and body began with Meeren et al. (2005), who found that body and facial expressions may produce similar Stroop effects (Meeren et al., 2005). Other researchers have also begun using combined emotional stimuli of face and body posture in their studies. This is a challenge to the standard pattern of emotional expression while also emphasizing the role of body language in expressing and perceiving emotions (Aviezer et al., 2012).

In recent years, with the deepening of research in the field of nonverbal emotional cue processing, many researchers have found that body expressions can have an impact on individual facial expression processing. A study showed that emotional body posture affects the processing of facial expressions, especially when the emotions conveyed by the body suggest danger (Poyo Solanas et al., 2018). Some researchers have used four basic emotions to study the asynchronous effect of facial expressions on body expressions. The results indicated that the perception of affective facial expressions significantly influenced the categorization of body-based emotion, particularly for bodily expression of happiness. The findings show that facial expressions influence the recognition of bodily expressions, despite asynchrony (Zhang et al., 2019). In addition, it was previously believed that body expression only works when facial expressions are ambiguous. However, research has found that body expressions have the greatest effect on facial expression recognition among the two expressions of happiness and fear (De Gelder et al., 2015). More interestingly, a study using temporal visual integration methods to examine the temporal and spatial integration of facial and body expressions showed that strong integration occurs when they are presented synchronously, indicating that the integration between emotional faces and bodies may be more sensitive than previously assumed (Lecker et al., 2017).

Gesture is a rich information carrier, including not only emotional information transmission but also semantic information. For example, the ‘OK’ gesture means ‘OK’ in China and the United States, while it means ‘money’ in Japan and ‘nothing’ in France. For that reason, it is necessary to pay attention to the cross-cultural study of gestures when selecting gestural stimuli as experimental materials (Wood et al., 2019). It is precisely because people’s interpretations of gestures are more diverse than facial expressions that gesture cues are often complementary. Therefore, gesture cues have significant advantages over facial expressions when expressing some implicit meaning or inexpressible information (Trofatter et al., 2015). Another study explored the effect of ambiguous faces on the semantic understanding of gestures through ERPs, and the results showed that gestures facilitated the understanding of emotional information when face processing resources were scarce (Proverbio et al., 2018).

Studies have shown that people with HSA pay more attention to the hands that express their physical emotions than people with LSA, possibly to avoid eye contact (Liedtke et al., 2018). Furthermore, people of different genders show significant differences in response times when recognizing complex face and gesture stimuli (Wood et al., 2019), which may be related to individual attentional characteristics and personality traits, such as those of people with SAD and anxiety disorders. At present, most of the studies on the processing of nonverbal affective cues are about faces, while the processing characteristics and neural basis of “gestures” as a neglected cue in emotion recognition remain to be explored. In recent years, increasing attention has been given to physical stimuli and combined stimuli. Incorporating body expressions, especially gestures, into emotional processing is important for adapting social situations and interactive behaviors in people with SAD.

Combining the shortcomings of previous studies with a more ecological perspective, we use video material of face-gesture combined emotional stimuli to explore the following two questions: Is there a dominant processing of compound emotional stimuli in individuals with HSA? Does gestural expressive information have a greater effect on emotion recognition in individuals with HSA? Therefore, the following research hypotheses can be obtained: (1) People with HSA have an emotionally congruent effect on combined expression stimuli recognition. (2) There is reversible interference between the processing of gestures and facial expressions. Specifically, the influence of gestures on facial expressions is greater than that of faces on gestures, and this difference is more significant in the HSA group.

## 2. Materials and methods

### 2.1. Participants

G* Power 3.1.9.7 was used for computation of the sample size. With reference to Cohen’s (1988) study, we set a medium effect size F (the value is 0.25), given the α value (0.05) and power value (0.95), and a total sample size of 36, i.e., at least 18 people in each social anxiety group. We recruited 150 participants via online advertising portals (e.g., Bulletin Board System) in the College of Education, Wenzhou University. Inclusion criteria were Asian ethnicity, young adults aged 18–23 years (to avoid the effect of age-related changes in vision and cognition that may affect task performance), and normal or corrected-to-normal vision. All participants were right-handed, had no mental illness, and had no previous medical history. Participants were offered 1 hour of course credit or cash in return for their time. The demographic information and questionnaire scores of all participants are shown in Table 1.

**Table 1:** Demographic information and scale scores of the HSA and LSA groups (*n*, *M* ± SD).

	HSA (*n* = 39)	LSA (*n* = 40)	*t/χ^2^*	*p*
Female	35	33	0.863	0.353
Average age	19.77 ± 1.307	19.85 ± 1.442	0.261	0.508
LSAS score	82.44 ± 15.04	27.45 ± 11.346	−18.305	<0.001
Trait anxiety score	48.17 ± 7.560	37.40 ± 7.096	−6.531	<0.001

Participants were asked to complete two questionnaires to measure psychopathology. Participants completed the Chinese version of the Liebowitz Social Anxiety Scale (LSAS, Liebowitz et al., 1987; He and Zhang, 2004) and the Trait Anxiety Inventory (T-AI, King et al., 1976). Groups were defined according to the total LSAS score. Finally, 39 people (35 women and 4 men) with an LSAS score higher than 70 (above the 73th percentile) were assigned to the HSA group, and 40 people (33 women and 7 men) with a score lower than 40 (below the 27th percentile) were assigned to the LSA group, with a total of 79 participants (*M* = 19.62 years, SD = 1.288 years). Independent sample t tests and chi-square tests revealed no significant difference in age and gender between the HSA and LSA groups (*p* > 0.05), and the LSAS and Trait Anxiety Inventory (T-AI) scores were significantly different between the two groups (*p* < 0.001). Ethical approval was provided by the Wenzhou University Ethics Committee. Participants provided written informed consent.

### 2.2. Questionnaires

The Liebowitz Social Anxiety Scale (LSAS, Liebowitz et al., 1987) is the most applicable instrument for assessing social anxiety, including clinical and self-report forms. It has 24 items and measures the rate of fear and avoidance of functional and social interaction situations on a 4-point Likert scale. The fear scale assesses levels of intensity ranging from 0 (none) to 3 (severe fear), while the avoidance scale assesses frequency of avoidance ranging from 0 (never) to 3 (usually). The total score was created by summing all the fear and avoidance scores, with higher scores reflecting more severe social anxiety symptoms. The validity of its self-report form has been supported in several studies (Safren et al., 1999; Baker et al., 2002; Mennin et al., 2002). The Chinese version of the LSAS has been validated in previous studies with good reliability and validity (He and Zhang, 2004). The Cronbach’s α of the LSAS in this study was 0.961.

Trait-anxiety was also measured to distinguish and evaluate personality trait anxiety and provide a more informed characterization of the sample. To achieve this, the State–Trait Anxiety Inventory (King et al., 1976) was used. It has a total of 40 items, 20 for trait anxiety measurement. Ratings are made on a 4-point Likert scale from 0 (never) to 3 (almost always). The trait version of the STAI has been found to have good convergent, discriminant, and construct validity and reliability (Bieling et al., 1998). Trait anxiety ranged from 0 to 67 (*M* = 44.99, SD = 6.68). The Cronbach’s α of the STAI in this study was 0.733.

### 2.3. Experimental stimuli and apparatus

**Figure 1:**
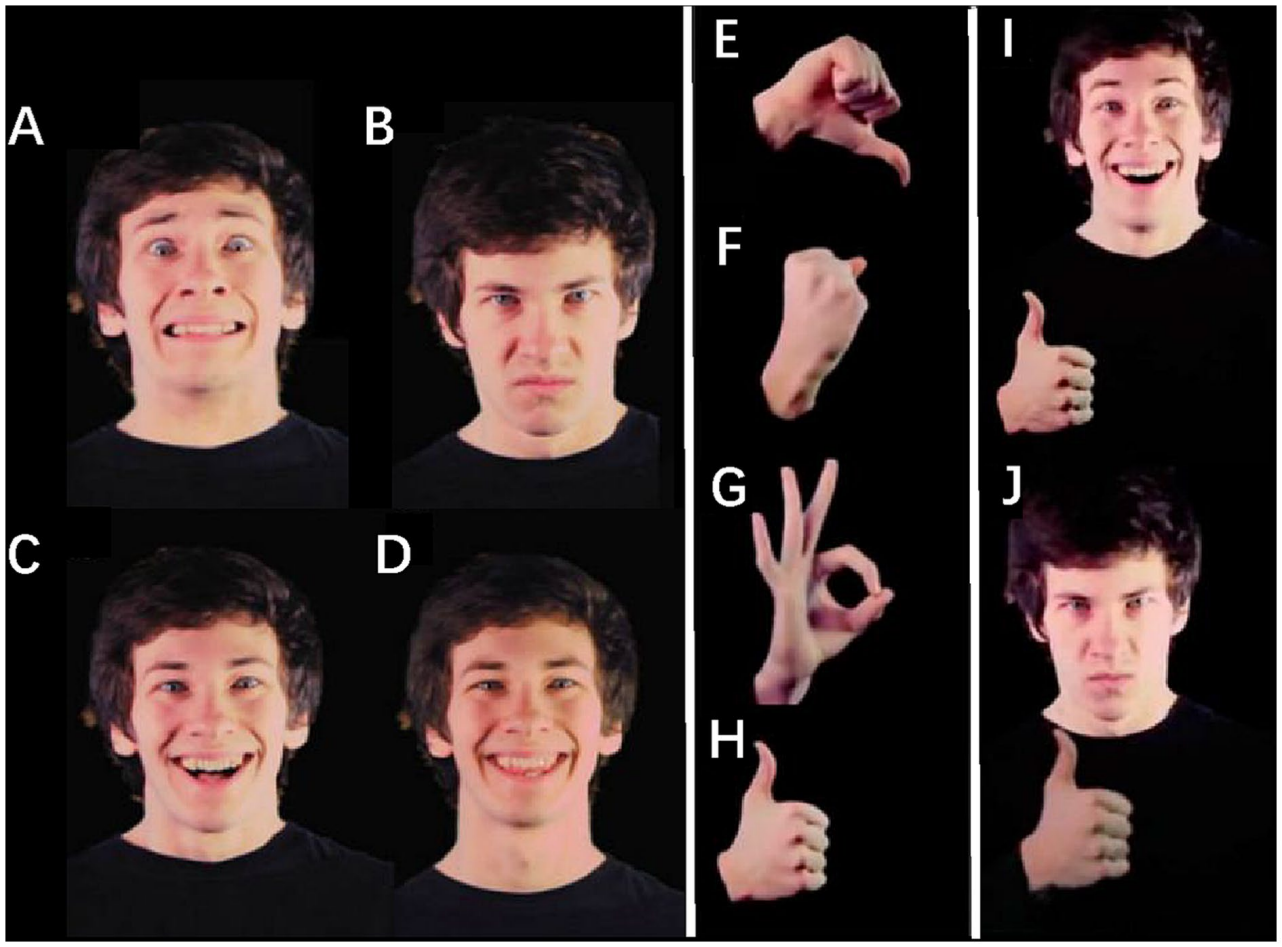
Still frames from Actor 1’s video stimuli. The dynamic facial expressions began from neutral and ended at the apex of the expression. We used 2 negative facial expressions, frightened **(A)** and angry **(B)**, and 2 positive expressions, excited **(C)** and happy **(D)**. For the dynamic hand gestures, all actors started with their hand off-camera, then raised it, emphasized the gesture with 2 pulses, and held it still for the remainder of the video. The negative gestures were thumbs-down **(E)** and fist **(F)**, and the positive gestures were A-OK **(G)** and thumbs-up **(H)**. Sample congruent **(I)** and incongruent **(J)** stimuli for the face-hand phase are also shown. Adapted from Wood et al. (2019).

The video stimuli were derived from online files made by Wood et al. (2019), and the experiment and stimuli files are available online.^1^ The video stimuli were taken by four white actors (two female, two male). We chose happy and excited emotions as positive facial expressions, while frightened and angry acted as negative facial expressions. The hand gestures were thumbs-up, A-OK, thumbs-down, and a fist raised as if in anger (see Figure 1). The dynamic facial expressions began from neutral and ended at the apex of the expression. The dynamic hand gestures began with the hand off-camera, then the actor raised their hand, emphasized the gesture with 2 superimposed beats (i.e., pulses), and held the position still for the remainder of the video (Wood et al., 2019). “Congruent” face-gesture pairs were combinations of positive facial expressions and positive hand gestures or negative facial expressions and negative hand gestures. “Incongruent” face-gesture pairs were combinations of a negative (either face or hand) stimulus and a positive (hand or face, respectively) stimulus. For the single stimuli phase, we also used gesture-only (16 in total) and face-only (16 in total) videos from each actor. The face-only stimuli showed the actors’ heads (with hands not visible), and the gesture-only stimuli showed the actors’ hands (with faces not visible).

Experimental materials were evaluated by 30 college students from Wenzhou University who did not participate in the formal experimental process. Both pleasantness and arousal were scored on a 9-point scale ranging from 1 to 9, with 1 representing the lowest degree and 9 representing the highest degree. A total of 32 videos with 16 facial expression stimuli and 16 gesture stimuli were statistically analysed. Independent sample *T* tests showed that the pleasantness of negative facial expression videos was significantly lower than that of positive facial expressions (*t* (58) =19.079, *p*<0.001, *d* = 4.926). The pleasantness of the negative gesture video was significantly lower than that of the positive gesture video (*t* (58) = 10.668, *p* < 0.001, *d* = 2.755). There was no significant difference in arousal between negative and positive gesture expression videos (*t* (58) = 0.656, *p* > 0.05, *d* = 0.169), while the arousal between negative and positive facial expression videos had a significant difference (*t* (58) = 2.750, *p* < 0.05, *d* = 0.551) (Table 2).

**Table 2:** Evaluation results of different emotional facial and gestures materials (*n*, *M* ± SD).

Valence	Video type		Pleasantness	Arousal
Positive	Facial gesture	Happy	6.80 ± 0.94	5.96 ± 1.05
		Excited	7.15 ± 1.98	6.69 ± 0.90
		Thumb up	6.96 ± 1.02	6.23 ± 1.20
		OK	6.41 ± 1.15	5.33 ± 1.44
Negative	Facial gesture	Anger	2.97 ± 0.82	5.23 ± 1.48
		Frightened	2.91 ± 0.85	5.64 ± 1.73
		Thumb down	2.63 ± 1.07	5.51 ± 1.57
		Fist	4.78 ± 1.78	5.56 ± 1.41

The experiment was conducted in a room with good sound insulation, soft light, and a comfortable temperature. Stimuli were programmed using PsychoPy 3.0 (Jonathan Peirce, University of Nottingham, United Kingdom). All participants sat during the computer task (viewing distance = 44 cm; vertical viewing angle = 19.9°; horizontal viewing angle = 30.8°). The faces and gestures of the characters in the experimental materials were presented in fixed positions on a liquid crystal display monitor (Dell: 17-inch LCD, resolution 1,024 × 768, refresh rate of 60 Hz; i7-6,700 processor; solid-state hard disk 120 GB + 1 TB mechanical hard disk; independent graphics card 1,070 GTX; 16 GB memory), and the background was set to gray (RGB was 128 × 128 × 128).

### 2.4. Procedure and design

A three-factor mixed design of 2 (group: HSA and LSA) × 2 (emotional valence: positive and negative) × 2 (task type: face and gesture) was adopted. The accuracy rate (%) and response time (ms) of the participants’ key responses were recorded.

Participants completed the demographic questionnaire, LSAS, T-AI, and then the emotional valence discrimination experiment. The experimental process includes a practice experiment and a formal experiment. The practice experiment consisted of 12 trials, and the experimental stimuli were four kinds of facial expressions and gestures. In each trial, participants were presented with a gray screen for 1,500 ms followed by a facial or gesture video that was presented for 4,000 ms each. Participants were assigned two tasks: one was to judge emotions based on facial expressions, and the other was to judge emotions based on gestures. The order of presentation between the two tasks was balanced within subjects. According to the instructions, the participants were supposed to press the ‘F’ key when judging the emotion of face/gesture as positive, while as negative, they pressed the ‘J’ key (adjusted according to the left or right handedness of the participants).

**Figure 2:**
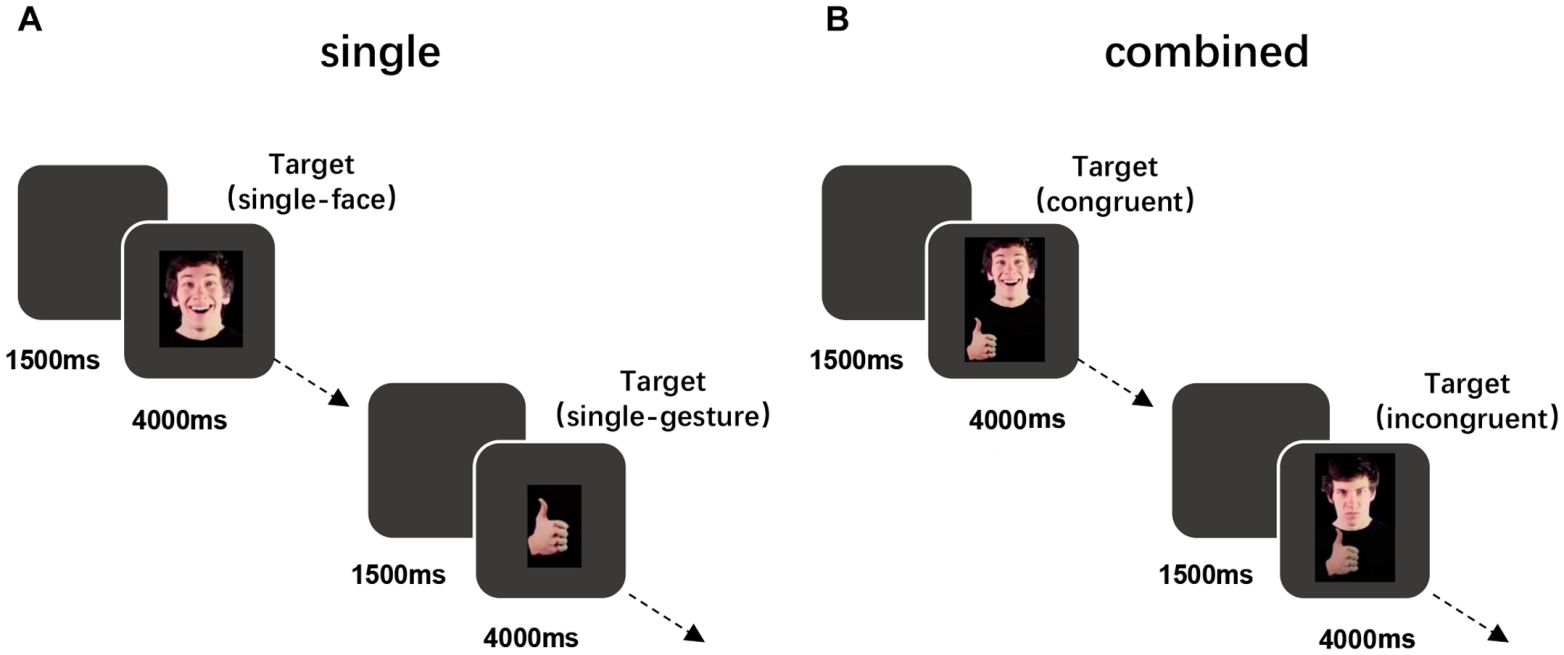
Schematic of trials in the single video stage **(A)** and combined video stage **(B)**. **(A)** Example of positive trials in which participants viewed a single positive face or gesture. **(B)** Example of positive-gesture trials in which participants viewed a positive gesture paired with a positive face (congruent) or a negative face (incongruent). See the online article for the color version of this figure. Adapted from Wood et al. (2019).

The formal experiment consisted of a single phase and a combined phase. In the single phase, 28 trials were presented randomly (16 trials of a single face and 12 trials of a single gesture). Participants could take a break after completing the single-phase task. The combined-stimulus phase was divided into two blocks, with appropriate rest between each block. There were 112 trials in this experimental phase, including face-target tasks and gesture-target tasks. There are 28 stimuli for each of the two tasks, and each stimulus is presented twice. The procedure is shown in Figure 2.

### 2.5. Statistical analysis

Accuracy (ACC) and reaction time (RT) for performance of the emotional valence discrimination experiment in both single and combined phases were collected. Data were imported into Excel for preprocessing. SPSS 25.0 (IBM, Somers, United States) statistical software was used for statistical analysis. Independent sample T tests were used to analyze the questionnaire scores of the two groups. Independent sample T tests were used to analyze the pleasantness and arousal of stimuli of two different valences (positive and negative). To explore the potential differences in participants’ performance at different task stages, we conducted 2 × 2 × 2 repeated measures analyses of variance (ANOVAs) for behavioral data with the group (HSA and LSA) as between-subject factors and the task stage (single and combined) and the task type (face and gesture) as within-subject factors. Then, we focused on the combined stage and conducted 2 × 2 × 2 repeated measures analyses of variance (ANOVAs) with the group (HSA and LSA) as between-subject factors and the task type (face and gesture) and emotional valence (positive and negative) as within-subject factors. At last, we divided the emotional valence into two conditions (congruent and incongruent) and conducted another ANOVAs with the task type (face and gesture) and congruency (congruent and incongruent) as within-subject factors.

## 3. Results

**Figure 3:**
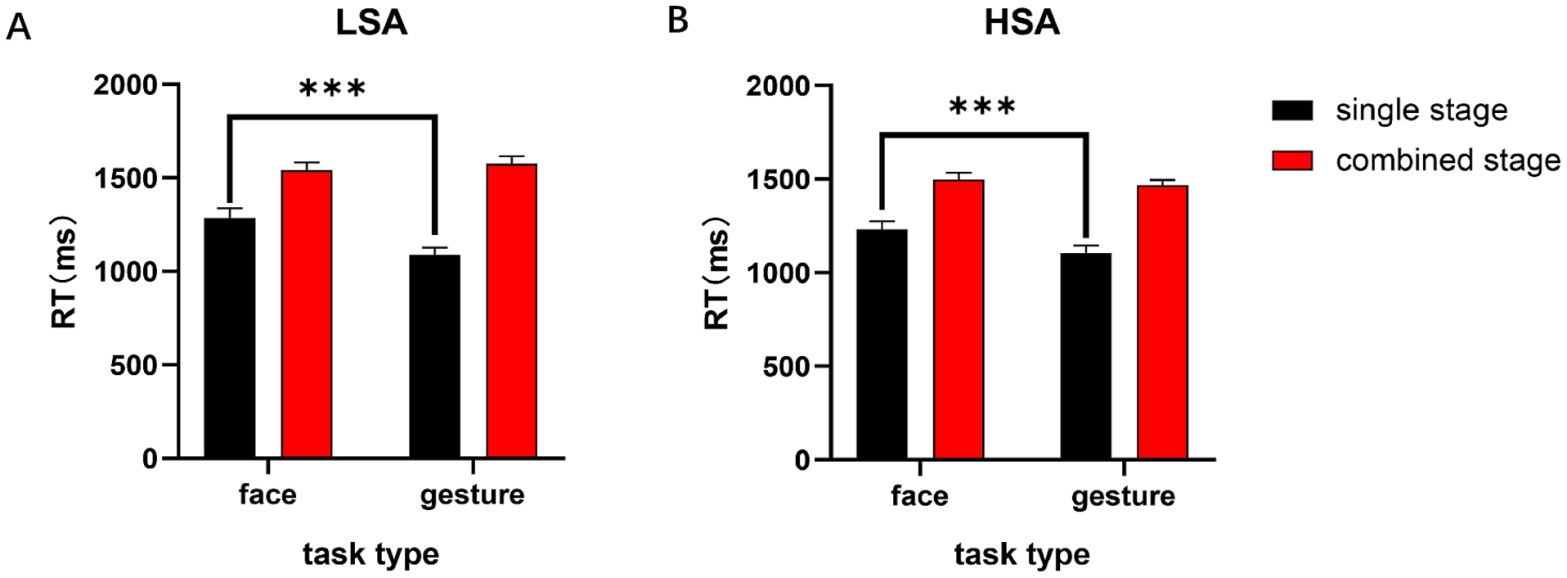
RTs of different stages and tasks in both the LSA and HSA groups. **(A)** RTs of facial and gestural expressions in single and combined stages in the LSA group. **(B)** RTs of facial and gestural expressions in single and combined stages in the HSA group. Error bars represent the mean SE. ***p < 0.001.

SPSS 25.0 was used for statistical analysis. Trials that were 3 standard deviations higher than average or faster than 150 milliseconds were excluded. We first conducted a 2 × 2 × 2 three-factor repeated-measures ANOVA with two tasks (face and gesture) and two stages (single and combined) as between-subject factors and found significant interactions between groups, task types and task stages (*F* (1, 77) =3.959, *p* = 0.050, *η*^2^ = 0.049). It was found that the main effect of task type was significant (*F* (1, 77) = 11.033, *p* = 0.001, *η*^2^ = 0.125), with the RT of the gesture (1309.33 ± 18.68) being significantly shorter than that of the face (1388.54 ± 29.29); the main effect of the task stage was significant (*F* (1, 77) = 208.242, *p* < 0.001, *η*^2^ = 0.73), and the RTs to the single stage (1177.24 ± 29.52) were significantly shorter than those to the combined stage (1520.61 ± 18.29), indicating that the RT of the stimuli-combined presentation was prolonged relative to the face and gesture presented alone. Furthermore, the interaction between task type and task stage was also significant (*F* (1, 77) = 22.31, *p* < 0.001, *η*^2^ = 0.225), and a simple effect analysis showed that in the single stage, the RTs of facial stimuli were significantly longer than those of gesture stimuli (*p* < 0.001). In the combined stage, there was no significant difference in facial and gesture processing (*p* > 0.05) (see Figure 3). The difference in accuracy of the subjects was not significant (*Ps* > 0.05). Descriptive statistical analysis is shown in Table 3.

**Table 3:** Reaction times (RT) and accuracy (ACC) of both single and combined emotional stimuli in the HSA and LSA groups (*M* ± SD).

Group	Type-stimuli	Reaction time (ms)	Accuracy (%)
HSA (*n* = 39)	Single-face	1236.65 ± 237.22	97.33 ± 4.70
	Combined-face	1506.54 ± 218.92	94.73 ± 5.37
	Single-gesture	1111.13 ± 243.76	79.89 ± 10.55
	Combined-gestures	1455.19 ± 159.76	86.81 ± 15.32
LSA (*n* = 40)	Single-face	1276.53 ± 338.72	97.47 ± 4.74
	Combined-face	1532.40 ± 260.26	92.94 ± 7.68
	Single-gesture	1084.10 ± 240.30	79.02 ± 10.26
	Combined-gestures	1584.42 ± 257.01	83.25 ± 13.66

**Figure 4:**
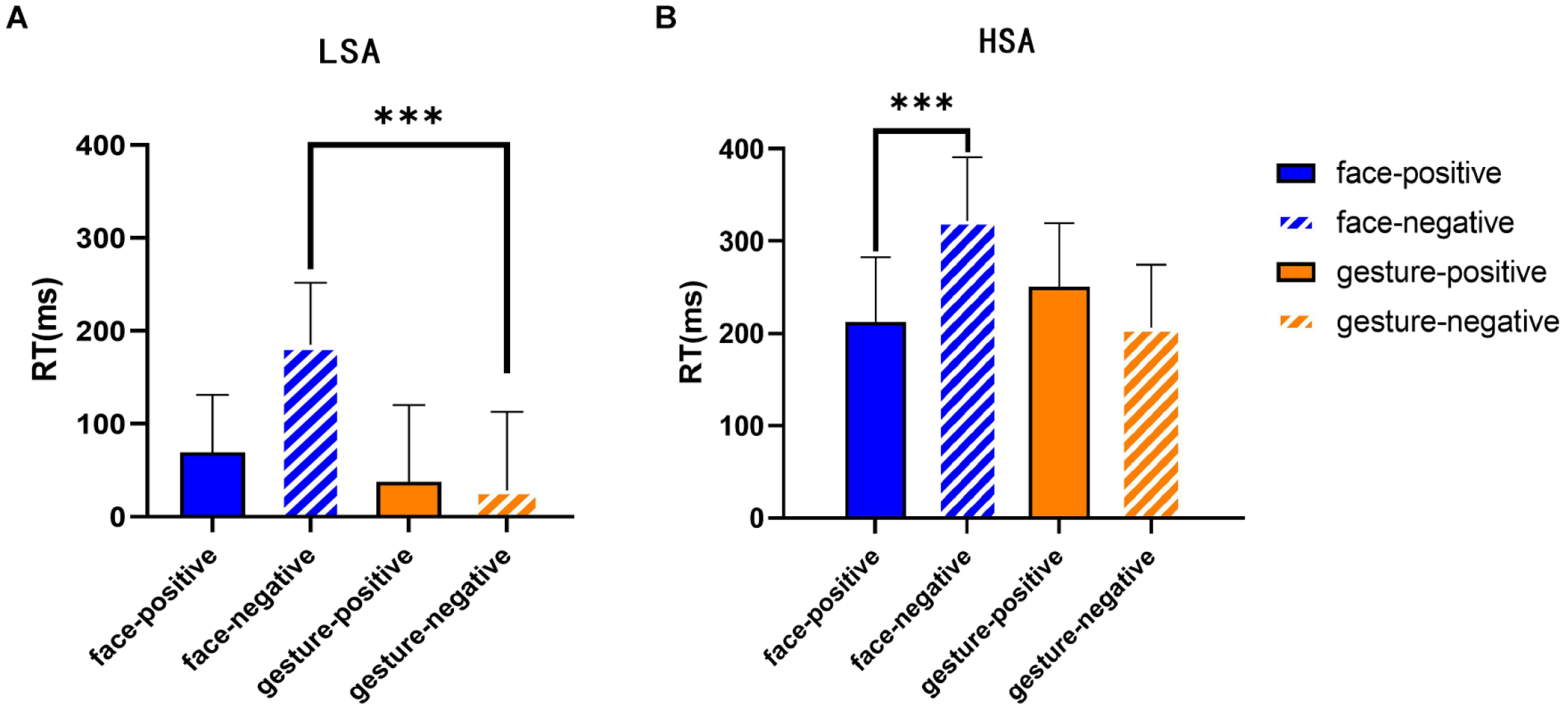
RTs of different expressions with different emotional valences. **(A)** RTs of facial and gestural expressions with different emotional valences in the LSA group. **(B)** RTs of facial and gestural expressions with different emotional valences in the HSA group. Error bars represent the mean SE. ^***^p < 0.001.

Due to the dynamic nature of the stimulus, there are differences in the start and end times of different facial expressions and gesture videos, which may lead to systematic errors in response time (RT). To eliminate those differences, the study calculated the average RT for each subject at a single phase for each hand or facial stimulus. The RT of the face-gesture phase was then adjusted by subtracting the mean single RT of the relevant subject and stimulus. The data were first subjected to a three-way mixed ANOVA of 2 (emotion valence: positive, negative) × 2 (task type: face, gesture) × 2 (group: HSA and LSA). It was found that the main effect of group was approaching significance (*F* (1, 77) = 3.669, *p* = 0.059, *η*^2^ = 0.045), with the RT of the HSA group (252.69 ± 339.66) being significantly shorter than that of the LSA group (53.22 ± 503.95); the main effect of emotion type was significant (*F* (1, 77) = 29.261, *p* < 0.001, *η* ^2^ = 0.275), and RTs for positive emotional stimuli (126.36 ± 472.04) were significantly shorter than those for negative emotions (179.55 ± 470.57). However, there was a significant interaction between task type and emotion type (*F* (1, 77) = 143.586, *p* < 0.001, *η*^2^ = 0.651), and a simple effect analysis showed that when the emotion type was positive, the difference in RT between face and gesture stimuli was not significant; when the emotion type was negative, the RT for gesture stimuli was significantly shorter than the face stimuli (*F* (1, 77) = 6.226, *p* = 0.015, *η*^2^ = 0.075) (see Figure 4), indicating that there was an attentional enhancement in subjects’ processing of gestures, and this attentional bias was more pronounced in the processing of negative gestures. Other main effects and interactions were not found (*Ps* > 0.05). Descriptive statistical analyses are presented in Table 4. None of the results for ACC were significant (*Ps* > 0.05), and subjects’ ACC was above 90% in all conditions.

**Table 4:** Reaction times (RTs) and accuracy (ACC) of both face and gesture emotional stimuli of different valences in the HSA and LSA groups (*M* ± SD).

Group	Type-valence	RT (ms)	ACC (%)
HSA (*n* = 39)	Face-Positive	212.08 ± 439.98	94.75 ± 6.39
	Face-Negative	330.09 ± 438.34	94.71 ± 7.16
	Gesture-Positive	257.08 ± 439.51	96.31 ± 11.88
	Gesture-Negative	211.50 ± 436.82	96.47 ± 9.04
LSA (*n* = 40)	Face-Positive	−1.330 ± 487.54	93.73 ± 6.93
	Face-Negative	148.43 ± 472.59	92.11 ± 10.35
	Gesture-Positive	37.61 ± 521.13	95.46 ± 11.75
	Gesture-Negative	28.16 ± 534.52	95.00 ± 11.60

**Figure 5:**
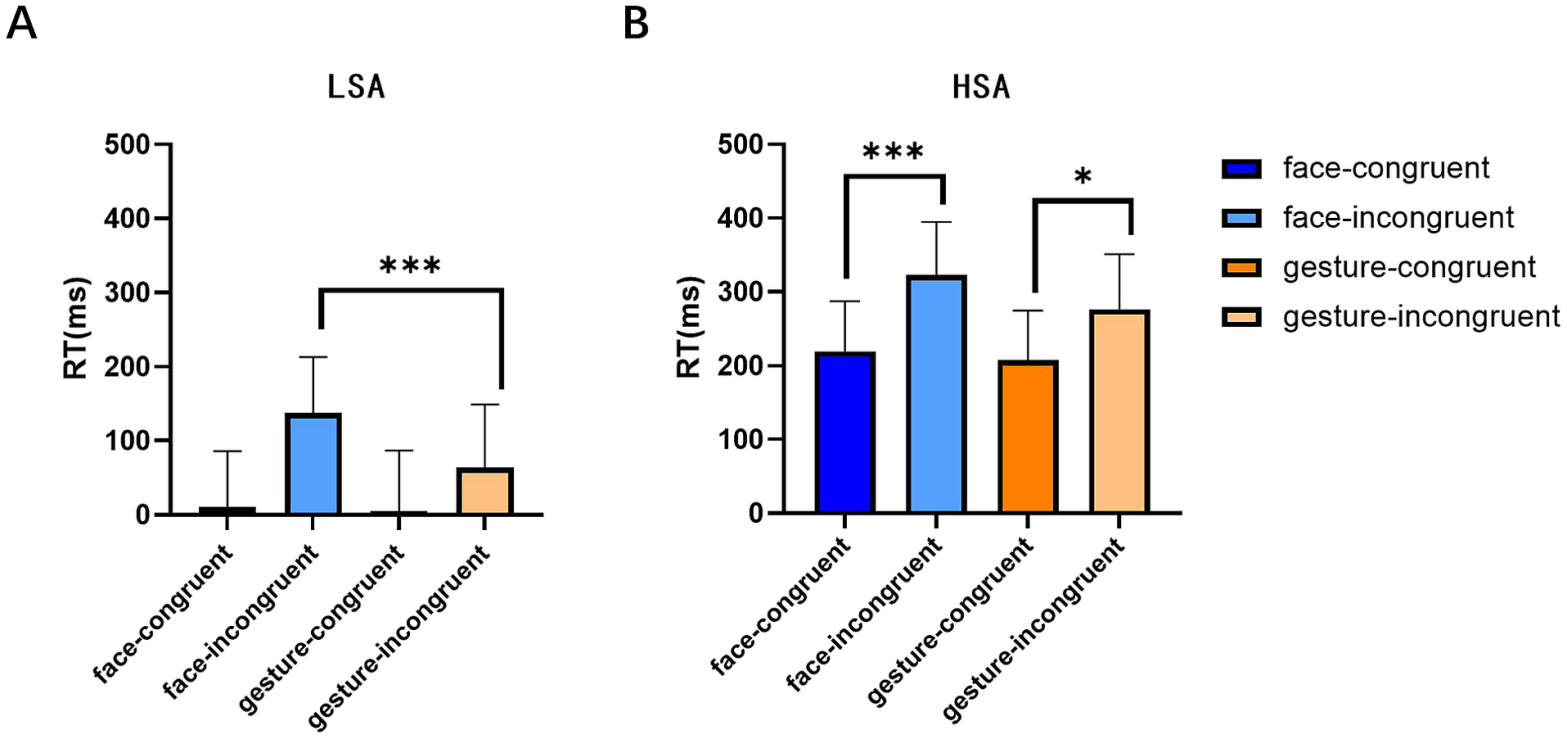
RTs of different expressions under different conditions in both the LSA and HSA groups. **(A)** RTs of facial and gestural expressions under congruent and incongruent conditions in the LSA group. **(B)** RTs of facial and gestural expressions under congruent and incongruent conditions in the HSA group. Error bars represent the mean SE. ^*^p < 0.05, ^***^p < 0.001.

From the perspective of emotion congruency of face and gesture, the data were then subjected to a three-way mixed ANOVA of 2 (congruency type: congruent, incongruent) × 2 (task type: face, gesture) × 2 (group: HSA and LSA). From the results of RTs, it was found that the main effect of group was marginally significant (*F* (1, 77) = 3.779, *p* = 0.056, *η*^2^ = 0.047), with the RT of the LSA (54.03 ± 503.97) being significantly shorter than that of the HSA group (256.57 ± 439.37); the main effect of the congruency type was significant (*F* (1, 77) = 61.604, *p* < 0.001, *η*^2^ = 0.444), and the RTs to congruent stimuli (110.56 ± 460.38) were significantly shorter than those to incongruent stimuli (200.04 ± 482.96). The interaction between task type and congruency type was significant (*F* (1, 77) = 13.448, *p* < 0.001, *η*^2^ = 0.664), and a simple effect analysis showed that under congruent conditions, there was no significant difference in facial and gesture processing (*F* (1, 77) = 0.257, *p* = 0.614, *η*^2^ = 0.003). Under incongruent conditions, the RTs of facial stimuli were significantly longer than those of gesture stimuli, indicating that compared to the influence of incongruent faces on gestures, incongruent gestures had a greater impact on facial expression processing (*F* (1, 77) = 7.481, *p* < 0.01, *η*^2^ = 0.089). Both in facial and gesture conditions, the RT of congruent stimuli was significantly shorter than that of incongruent stimuli (*F* (1, 77) = 104.984, *p* < 0.001, *η*^2^ = 0.577; *F* (1, 77) = 17.691, *p* < 0.001, *η*^2^ = 0.187), as shown in Figure 5. None of the results for ACC were significant (*Ps* > 0.05), and subjects’ ACC was above 90% in all conditions (see Table 5).

**Table 5:** Reaction times (RTs) and accuracy (ACC) of both face and gesture emotional stimuli in different conditions in the HSA and LSA groups (*M* ± SD).

Group	Type-condition	RT (ms)	ACC (%)
HSA (*n* = 39)	Face-Congruent	219.55 ± 423.19	97.74 ± 3.56
	Face-Incongruent	322.59 ± 450.89	91.72 ± 9.27
	Gesture-Congruent	207.47 ± 419.33	99.14 ± 2.56
	Gesture-Incongruent	276.65 ± 464.06	93.58 ± 20.27
LSA (*n* = 40)	Face-Congruent	10.079 ± 481.49	97.38 ± 3.50
	Face-Incongruent	137.10 ± 479.53	88.41 ± 14.61
	Gesture-Congruent	5.139 ± 517.50	98.12 ± 5.16
	Gesture-Incongruent	63.81 ± 537.37	92.50 ± 20.04

## 4. Discussion

The findings of this study, which investigated how individuals with social anxiety recognize facial expressions and gestures using a behavioral experiment, revealed that participants responded faster to gestures than faces when the stimulus was presented alone (single stage). However, when a combined gesture-face stimulus was presented, there was no significant difference between the two in both the high social anxiety (HSA) and low social anxiety (LSA) groups. The results also showed that participants responded more quickly and attentively to negative gestures but slower to negative faces, indicating two distinct types of attentional bias - attentional enhancement and attentional fixation - for different task types. This suggests that cognitive emotion processing may differ between faces and gestures. Additionally, the findings demonstrated that when faced with incongruent situations, participants processed gestures faster than faces, suggesting that incongruent gestures had a stronger impact on face processing than incongruent faces on gestures.

Regarding processing for a single task type (face or gesture), the recognition time of facial expressions was longer than that of gestures, contrary to the view by previous scholars that face recognition has more advantages (Wang, 2021). This implies that facial expressions may not be the most direct and rapid cue for judging emotions. In this study, hand gestures were recognized more quickly than facial expressions, supporting the idea of asynchronous effects of body posture on emotion recognition.

Upon examining the processing of combined face-gesture stimuli, it was found that the inclusion of combined stimuli seemed to increase the participants’ cognitive load, resulting in significantly longer response times compared to the single phase. Interestingly, we discovered that participants had quicker and more attentive responses to negative gestures, while responses to negative faces were slower. This suggests the presence of two distinct types of attentional bias - attentional enhancement and attentional fixation - for different types of tasks. Previous studies (Tang, 2018; Kong et al., 2022) have shown that individuals with high social anxiety (HSA) tend to develop a bias toward negative faces, especially those that are threatening. This bias may be due to inadequate information processing by individuals with social anxiety (Zimmer-Gembeck and Nesdale, 2013) or a lack of understanding of the implied meaning of the stimuli. Consequently, individuals with HSA may exhibit increased or fixed attention toward negative gestures or facial expressions. However, in our experiment, individuals with HSA did display some degree of bias in processing negative cues (particularly toward gesture cues), but the difference compared to individuals with low social anxiety (LSA) was not statistically significant. We speculate that this could be attributed to the limited sample size of our study and the potential interference of other factors, such as individual differences or stimulus material, which may have influenced the experimental results.

Further analysis of the congruency effect for both types of stimuli revealed that response times in the emotion-incongruent condition were significantly longer than those in the congruent condition for both the face-target and gesture-target tasks. This aligns with previous studies on the emotional congruency effect of the visual channel, where nontarget stimuli sharing similar emotional valence can somewhat alleviate the participants’ cognitive load and enhance the recognition of target stimuli (Kret et al., 2013). Additionally, response times in the face-target task were significantly longer than those in the gesture-target task in the incongruent condition, indicating a heightened emotion-conflict effect associated with incongruent gestural contextual cues on facial expression recognition during cognitive processing. In other words, gesture cues have a greater influence on face processing, while facial expression cues have less influence on gesture processing. The findings of this study somewhat align with the second view in the ongoing debate on the influence of facial expressions and gesture cues in emotional cue processing. Previous studies have provided divergent interpretations, with some supporting the notion that facial expressions influence gesture expression processing and others arguing that gesture cues dominate in influencing facial expression processing.

The study revealed significant individual differences in the interpretation of gestural stimulus materials. For example, the validity of the gesture “fist” was rated as neutral rather than negative, and its ACC was less than 30% of the total “fist-target” trials in the formal experiment. This suggests that different individuals had different interpretations of the “fist” gesture, leading to its exclusion from the final results. Interestingly, some subjects perceived the fist gesture not as an aggressive gesture but as a signal of encouragement toward their friends. In a related study that investigated the relationship between gesture comprehension and semantic brain regions using homemade gesture picture materials, in which the researchers described the corresponding statement of “clenched fist” as “look how strong I am,” the results showed significant congruency between gesture understanding and the description (Proverbio et al., 2018). Therefore, in this study, the subjects perceived “fist” as “cheer,” probably because they thought the gesture could convey some power and thus judged it as a positive gesture. Since the average recognition results of all subjects on fist gestures tend to be neutral, we suspect that at least half of the subjects consider the gesture to be negative. Another explanation for the above phenomenon is that gestures may be divided into two conditions: strength and powerlessness. Studies have found that the effect of gestures with strength on emotional face recognition was more pronounced. However, the dynamic gestures used in this study were apparently stronger in action amplitude than static fists, and the action amplitude of gestures could be appropriately reduced in the future to avoid subjects’ comprehension errors.

The study has several limitations. First, the lack of open-source localized emotional materials in China resulted in the use of facial stimuli from previous foreign research. This may have introduced the other-race effect, potentially affecting participants’ response time and leading to inaccurate experimental results. Furthermore, the dynamic nature of the materials meant that different participants had varying abilities to gather stimulus information, which could explain the large standard deviation in the data. Second, there were noticeable cultural differences in the interpretation of gesture materials. For instance, the “waving fist” action was perceived as a positive expression of cheering for the other person by a significant number of subjects. The performance in judging emotional gestures was clearly inferior to that for faces. Additionally, the differences in arousal between certain facial expressions may have influenced participants’ responses in the experiment. Future studies will explore the effects of positive and negative facial expression arousal on the HSA group. Last, although the Liebowitz Social Anxiety Scale and Trait Anxiety Inventory have been proven to be highly effective measurement tools, the reliability of online data collection methods remains poor.

## 5. Conclusion

The present study explored the attentional characteristics and mechanisms of the interaction between gesture processing and facial expression processing among university students with high and low social anxiety and reached the following conclusions: (1) There is a distinction in the processing of faces and gestures, with individuals recognizing gestures faster than faces; (2) There is an attentional enhancement in the processing of gestures, particularly for negative gestures; and (3) Emotional cues from facial expressions and gestures have a contextual effect on their processing. When the emotional valence of faces and gestures align, it facilitates the recognition of both. However, incongruent gestures have a stronger impact on the processing of facial expressions compared to facial expressions themselves, suggesting that the processing of facial emotions is more influenced by environmental cues provided by gestures.

## Data availability statement

The original contributions presented in the study are included in the article/supplementary material, further inquiries can be directed to the corresponding authors.

## Ethics statement

The studies involving humans were approved by Wenzhou University Ethics Committee. The studies were conducted in accordance with the local legislation and institutional requirements. The participants provided their written informed consent to participate in this study.

## Author contributions

XZ and YG carried out research and data analysis. LF and SX conceived the project and prepared the manuscript. TX and WL reproof the data and improved the manuscript. All authors contributed to the article and approved the submitted version.

## Funding

This work was sponsored by Zhejiang Provincial Natural Science Foundation of China grant no. LQ22C090003 awarded to LF.

## Conflict of interest

The authors declare that the research was conducted in the absence of any commercial or financial relationships that could be construed as a potential conflict of interest.

## Publisher’s note

All claims expressed in this article are solely those of the authors and do not necessarily represent those of their affiliated organizations, or those of the publisher, the editors and the reviewers. Any product that may be evaluated in this article, or claim that may be made by its manufacturer, is not guaranteed or endorsed by the publisher.

## Footnotes

^1^ https://osf.io/9xs48/
